# Lessons Learned for the Assessment of Children’s Pesticide Exposure: Critical Sampling and Analytical Issues for Future Studies

**DOI:** 10.1289/ehp.7674

**Published:** 2005-06-24

**Authors:** Richard A. Fenske, Asa Bradman, Robin M. Whyatt, Mary S. Wolff, Dana B. Barr

**Affiliations:** 1 Department of Environmental and Occupational Health Sciences, School of Public Health and Community Medicine, University of Washington, Seattle, Washington, USA; 2 Center for Children’s Environmental Health Research, School of Public Health, University of California, Berkeley, California, USA; 3 Columbia Center for Children’s Environmental Health, Mailman School of Public Health, Columbia University, New York, USA; 4 Department of Community and Preventive Medicine, Mount Sinai School of Medicine, New York, New York, USA; 5 National Center for Environmental Health, Centers for Disease Control and Prevention, Atlanta, Georgia, USA

**Keywords:** children, exposure, GPS, organophosphates, pesticides

## Abstract

In this article we examine sampling strategies and analytical methods used in a series of recent studies of children’s exposure to pesticides that may prove useful in the design and implementation of the National Children’s Study. We focus primarily on the experiences of four of the National Institute of Environmental Health Sciences/U.S. Environmental Protection Agency/ Children’s Centers and include University of Washington studies that predated these centers. These studies have measured maternal exposures, perinatal exposures, infant and toddler exposures, and exposure among young children through biologic monitoring, personal sampling, and environmental monitoring. Biologic monitoring appears to be the best available method for assessment of children’s exposure to pesticides, with some limitations. It is likely that a combination of biomarkers, environmental measurements, and questionnaires will be needed after careful consideration of the specific hypotheses posed by investigators and the limitations of each exposure metric. The value of environmental measurements, such as surface and toy wipes and indoor air or house dust samples, deserves further investigation. Emphasis on personal rather than environmental sampling in conjunction with urine or blood sampling is likely to be most effective at classifying exposure. For infants and young children, ease of urine collection (possible for extended periods of time) may make these samples the best available approach to capturing exposure variability of nonpersistent pesticides; additional validation studies are needed. Saliva measurements of pesticides, if feasible, would overcome the limitations of urinary metabolite-based exposure analysis. Global positioning system technology appears promising in the delineation of children’s time–location patterns.

Accurate characterization of children’s exposure to pesticides has proven to be a particularly challenging aspect of the field of exposure assessment. First, the term “pesticides” encompasses a diverse array of chemicals that can potentially produce a wide variety of health effects. Second, exposure of children to pesticides can occur through multiple pathways and routes. For example, the U.S. Environmental Protection Agency (EPA) considers food, drinking water, and residential pesticide use all to represent important sources of exposure, and these exposures can occur simultaneously or sequentially through the routes of ingestion, inhalation, and dermal contact (Cohen Hubal et al. 2000). Certain subpopulations, such as children living in agricultural communities or children whose parents work with pesticides, may be exposed through additional pathways. Third, many pesticides have short residence times in the body, making it difficult to characterize exposures from biologic samples. Finally, chemical exposures may have substantially different health consequences for children depending on the developmental stage during which the exposure occurs, requiring exposure characterization at multiple time points.

Our purpose in this article is to examine sampling strategies and analytical methods associated with a series of recent population studies that have sought to characterize children’s pesticide exposure, and to distill from these experiences a number of lessons learned. In this article, we focus primarily on the experiences of the National Institute of Environmental Health Sciences/U.S. EPA Children’s Centers located at Columbia University, the University of California at Berkeley, Mount Sinai Medical Center, and the University of Washington. We have also included a review of several University of Washington studies that predated establishment of the children’s centers and that were conducted under the auspices of the Pacific Northwest Agricultural Safety and Health (PNASH) Center, sponsored by the National Institute for Occupational Safety and Health, and the U.S. EPA Science To Achieve Results (STAR) Grant Program. This article is not meant to be an exhaustive review of exposure assessment methods, but rather a first-hand commentary on the use of particular methods in our studies. We therefore have not been able to include an analysis of a number of important studies conducted at other institutions, such as the Minnesota Children’s Pesticide Exposure Study (Adgate et al. 2001; Quackenboss et al. 2000) and studies of children’s exposure along the U.S.–Mexican border (U.S. EPA 2004).

In this article we first examine the rationale and methods of exposure data collection in the population studies and then review the substantial challenges associated with the analysis of pesticides in novel and complex matrices, and the interpretation of these analytical findings. It is our hope that experience gained from this work will prove useful to researchers embarking on longitudinal cohort studies, such as the proposed National Children’s Study.

## Sampling Strategies in Population Studies

Data used to construct exposure estimates or classifications can be drawn from a variety of sources, ranging from general information regarding pesticide use to personal measurements. Table 1 presents the approaches taken in the studies under review. The first two columns provide source information and environmental measurement methods; the remaining columns categorize various types of exposure samples collected according to age, because different sampling strategies are more or less practical and valuable within these time frames. Table 2 indicates the analytes measured in five biologic sample matrices collected in these studies.

### Pesticide source information.

Virtually all children’s exposure studies collect historical and contemporaneous information regarding pesticide use. In most cases, these data are collected through parental questionnaires or interviews and pertain to pesticides in and around the residence. In general, we have found that parents are best able to provide general information regarding the use of products (e.g., control of particular insects, control of weeds) but may not be able to provide detailed information on specific chemicals (Lu et al. 2004; Whyatt et al. 2002). In preliminary analyses of questionnaires administered by the Columbia center, women provided a pesticide product name for only 39% of the pest control methods reported to be used in the home during pregnancy and, in particular, were rarely able to identify the pesticide products used by an exterminator. Further, pesticide products can have the same brand name but contain different active ingredients, further complicating use of questionnaire data in pesticide exposure assessment.

Investigators for most of the reviewed studies have thus gone a step further to visually inspect the pesticide products in the home, sometimes referred to as a pesticide inventory. For example, study staff from the Berkeley center recorded the U.S. EPA registration number and the active ingredients on the label of each home pesticide. The registration number was later entered into a pesticide product database maintained by the California Department of Pesticide Regulation to confirm all active ingredients. Records of commercial pesticide applications can also be accessed during home visits (Berkowitz et al. 2003; Whyatt et al. 2003).

Identification of specific products can be very helpful in determining whether or not a particular class of chemicals has been used in the residence and may inform subsequent sampling plans, but the presence or absence of specific products does not generally enter into the development of an exposure metric for the residents. Frequency of residential pesticide use could be used potentially to sort children into exposure categories, but such an approach has not been fully validated. One study has shown that personal air levels of organophosphate (OP) pesticides were significantly higher among women who reported using exterminator sprays, can sprays, and/or pest bombs during pregnancy compared with those reporting no OP pesticide use (Whyatt et al. 2002, 2003). Another study demonstrated that children whose parents reported garden use of insecticides had higher levels of OP pesticide metabolites than did children whose parents did not use garden insecticides (Lu et al. 2001).

Food can be an important source of pesticide exposure for children, but most of the studies reviewed here have not devoted substantial resources to an evaluation of the dietary pathway. The Mount Sinai center obtained maternal prenatal dietary food frequency data during pregnancy only, with specific information about fish consumption. The Berkeley center also obtained a detailed prenatal food frequency questionnaire. Additional information was also obtained on fruit and vegetable consumption for the pregnant women and, later on, for their children. The Berkeley center and the PNASH center have collected duplicate diets from a relatively small number of children (Fenske et al. 2002a). Such an approach provides very useful quantitative information on exposure but is extremely time-consuming and expensive. A diet diary has also been used to distinguish children whose intake of fresh produce and juices was primarily organic and proved effective in classifying children’s OP pesticide exposure (Curl et al. 2003a).

Studies of children of agricultural workers have focused on potential paraoccupational exposure, collecting data on the transmission of pesticides from the workplace to the home by parents or other adult household members, as well as data on residential proximity to pesticide applications (Bradman et al. 1997; Curl et al. 2002; Eskenazi et al. 2003; Koch et al. 2002; Lu et al. 2000; Simcox et al. 1995). Results to date indicate that both of these pathways can contribute to children’s exposures in agricultural communities and would need to be considered in the design of a study that included rural populations. Studies at the Berkeley center have taken advantage of California’s unique Pesticide Use Reporting system, and researchers there are investigating the use of these data as predictors of pesticide exposure in their cohort (Castorina et al. 2003). The Washington center completed a 2-year intervention to reduce take-home exposure in 2002; the Berkeley center is currently conducting a similar intervention.

### Environmental monitoring.

House dust samples have been collected in most of the reviewed studies and have served as a reliable indicator of residential pesticide contamination (studies conducted at the PNASH Center), although not necessarily as a surrogate for children’s exposures (Curl et al. 2002; Fenske et al. 2002b; Lu et al. 2000; Simcox et al. 1995). A practical problem can arise when insufficient dust is available for analysis, as was the case for the Mount Sinai studies. In the Berkeley center studies, the average mass of 509 dust samples was 9 g/m2. The average of the fraction < 150 μm in diameter used for chemical analyses was 7 g/m2. About 20% of the samples had a fine fraction of < 0.5 g total. Most laboratory methods for pesticides require 0.5–2 g dust. It is likely that only a single chemical analysis will be possible for a significant fraction of homes, thus limiting future tests for other chemicals. The Berkeley, Mount Sinai, and PNASH centers have investigated alternate methods of measuring pesticide concentrations in child environments, such as indoor air and surface wipe sampling (Lu et al. 2004). A protocol that is currently being validated involves mailing study participants an alcohol wipe with instruction for wiping dust on the top of a specified doorframe. The sample is then placed in a resealable plastic bag and mailed back to the study team. Advantages include low cost of sample collection and low participant burden. However, research is currently ongoing to determine detection limits and detection frequencies using this method.

The Columbia center has conducted extensive indoor air sampling. For chlorpyrifos and diazinon, the correlation between 48-hr personal air samples collected from the mother during the third trimester and average 2-month indoor air levels over the final 2 months of pregnancy were strong (r > 0.7, p < 0.001) (Whyatt et al. 2003). Air and dust levels were not significantly correlated in a pilot study conducted by the Mount Sinai group; this may have been due to the very small amount of dust collectable in these homes (Markowitz S, personal communication). In addition to the OP pesticides several carbamates and pyrethroids have been measured in personal air samples collected from the mother over 48-hr during pregnancy (Whyatt et al. 2002, 2003).

Evidence of chemicals in a child’s environment does not necessarily provide the basis for a sound exposure metric. Dust, wipe, and indoor air measurements (including personal air samples) have not shown strong associations with biologic measurements (Curl et al. 2002; Whyatt et al. 2003). It is not clear whether the lack of strong associations is due to confounding factors (e.g., dietary exposure), to variability in the biologic measurements (including toxicokinetic considerations (discussed below), or to a relatively weak link between residential contamination and child exposures.

Environmental monitoring in these studies has focused almost exclusively on the home or residential setting and has not yet been extended to child care centers and schools. The Washington studies have included wipe sampling and dust sampling of commuter vehicles of workers to document the movement of agricultural pesticides from the workplace to the home (Curl et al. 2002; Lu et al. 2000).

### Hand wipe sampling.

Initial attempts to look at direct child exposures have included the use of hand wipes to collect pesticides from children’s hands. These methods include wiping the child’s hand with sterile gauze dressing pads that have been moistened with isopropanol, or asking the child to place a hand in a bag containing isopropanol (Bradman et al. 1997). Gordon et al. (1999) found excellent correlations between chlorpyrifos in indoor air and corresponding dermal wipes but poor correlations between chlorpyrifos in dust and dermal wipes. Another study reported weak associations between OP pesticide concentrations in hand wipes, house dust, and urinary levels of OP metabolites (Shalat et al. 2003). The Columbia center conducted hand wipes but found all samples to be less than the limit of detection.

### Clothing dosimeters.

Other techniques for assessing children’s dermal exposures include use of clothing dosimeters such as cotton gloves, union suits, and socks (Fenske 1993; Lewis 2005). The Berkeley center has experimented with clothing dosimeters in recent studies. Infants (children 6 and 12 months of age) wore precleaned cotton socks and union suits for several hours in their residential environments.

### Maternal exposure.

Personal air sampling has been used effectively to monitor maternal exposures during pregnancy by Columbia researchers (Whyatt et al. 2002, 2003). Investigators used motion detectors to determine whether or not the women complied with the request to carry the personal air monitors; motion detectors were installed in the backpacks of randomly selected women. Results were obtained from monitors worn by 113 women for approximately 48 hr each. For the average woman, nearly 95% of the total number of motion detections occurred during waking hours. In addition, 98% of the women self-reported that the air monitor was near them for least 40 of the 48 hr of the personal air monitoring.

This study (Whyatt et al. 2003) also found that levels of several OP and carbamate pesticides measured in the 48-hr personal air samples were significantly correlated with levels in 2-week indoor air samples, indicating that, at least for these pesticides, the 48-hr air samples provided a reasonable estimate of exposure over a longer period during pregnancy. In addition, there was little variability in indoor air levels of the insecticides, and the correlations between each of the insecticides in each of the 2-week air samples were highly significant. In cases where sampling bracketed an application event, it is likely that high levels would be observed initially, increasing temporal variability.

Blood samples have been collected throughout pregnancy to assess body burden of pesticides in the Berkeley, Columbia, and Mount Sinai center studies. No association was seen between insecticide levels in maternal blood collected at delivery and maternal self-reported pesticide use during pregnancy in one study (Whyatt et al. 2003). Weak correlations were seen between pesticide levels in the maternal personal air samples collected during pregnancy and in blood samples collected at delivery (r = 0.10–0.19). However, the correlations were generally stronger when analyses were restricted to women for whom the personal air sample was collected within a month of collection of the blood samples at delivery (r = 0.13–0.45). Maternal and umbilical blood insecticide levels (chlorpyrifos, diazinon, the propoxur metabolite 2-isopropoxyphenol, and bendiocarb) at delivery were highly correlated, indicating that the pesticides are readily transferred to the fetus during pregnancy. Significant inverse associations were seen between chlorpyrifos in umbilical cord blood and both birth weight and length, whereas no association was seen between chlorpyrifos in maternal personal air samples and the same measures of fetal growth (Whyatt et al. 2004). These results suggest that the biomarkers may better reflect exposure from all routes, not only the amount of insecticides absorbed by the mother but also the amount of the absorbed dose that has been transferred to the developing fetus (Whyatt et al. 2004).

Urine samples have also been collected from women during pregnancy in several studies. Investigators at the Berkeley center found that pesticide metabolites in samples collected in the first and third trimester were not correlated. Within-person variability was approximately two times higher than between-person variability, suggesting that more urine samples collected during pregnancy would improve exposure classification (Eskenazi et al. 2004). A moving estimate of the coefficient relating dimethyl OP metabolite levels to shorter gestation was used to show that exposures in later pregnancy may be associated with shorter pregnancies. Blood cholinesterase levels were inversely correlated with gestational duration, consistent with findings for dimethyl OP pesticide metabolites, although no significant correlation between blood cholinesterase and urinary metabolite levels was observed.

The Mount Sinai center collected urine samples in the third trimester of pregnancy and found that approximately 70% of the women in the cohort had been exposed to pesticides, but no associations were found between these biologic levels and pesticide questionnaire data (Berkowitz et al. 2003). In a preliminary analysis of data from the Columbia center, weak but significant correlations were seen between average chlorpyrifos and diazinon levels in indoor air samples collected over the final 2 months of pregnancy and their respective metabolites in urine samples collected biweekly from the mothers over the same time frame.

In summary, it is unlikely that questionnaire data alone can prove adequate for exposure classification of women during pregnancy. However, it appears that systematic monitoring through personal air sampling and biologic monitoring in combination with questionnaire data would yield useful exposure data for epidemiologic investigations.

### Perinatal exposure.

Several novel sampling methods are under development to determine perinatal exposure levels, including sampling of amniotic fluid, meconium, and cord blood. A pilot study from the Berkeley center of 100 amniotic fluid samples, slated for disposal after amniocentesis, were analyzed for a number of pesticides and their metabolites, including the OP pesticides (Bradman et al. 2003). Target analytes were detected with frequencies ranging from 5 to 70%. Levels were low compared with levels reported in urine, blood, and meconium. Because of risks to the fetus, amniotic fluid typically can be collected only when medically indicated amniocenteses are conducted, usually around 18–20 weeks of gestation, or during scheduled cesarean sections. Therefore, the population sampled will not necessarily be representative of a larger population of pregnant women. For women already undergoing this procedure, the collection of amniotic fluid for research purposes is noninvasive and causes no additional risk.

At the Columbia center, meconium samples were collected from 20 newborns and analyzed for OP pesticide metabolites (Whyatt and Barr 2001). Detection frequencies were very high for some of these analytes, but others were not detected. Metabolite levels were similar to those seen in adult urine in population-based research. Metabolites were stable at room temperature over 12 hr. These initial results indicate that the measurement of pesticide levels in meconium has promise as a bio-marker of prenatal exposure.

Cord blood has been sampled in three studies. Mount Sinai center investigators collected cord blood for enzyme, lead, and gene analyses. The Mount Sinai group relied on hospital staff for cord blood retrieval, with prenotification of impending delivery and a note on the chart, with the result that 59% of the participants’ cord blood was obtained. Columbia center investigators reported that successful collection of these samples required that a member of the research staff team follow the progress of the labor, go to the labor room before delivery to remind the delivery room staff that the woman is in the study, and assist with the sample collection. Umbilical cord blood was obtained by syringing the blood into heparinized syringes at the point the cord enters the placenta. To date, a cord blood sample has been obtained from 81% of the infants in the study. An average of 29 mL (range, 2–58 mL) was collected per delivery, with > 22 mL collected in 75% of deliveries and ≥30 mL collected in 50% of the deliveries (Whyatt et al. 2003). The Berkeley center investigators reported a similar proportion of cord blood samples collected and found that successful collection of cord blood required close cooperation with hospital staff to develop procedures that eliminated risks of inadvertent sticks (Eskenazi et al. 2003).

In summary, the perinatal sampling procedures described here are in the early stages of development and will need additional study and validation. However, they hold promise for collecting quantitative exposure data at a critical stage of child development.

### Infant and toddler exposure.

Traditional urine bags have been used in clinical settings and have proven useful for pesticide-related studies in children (Royster et al. 2002). The Berkeley center has been successful collecting urine from children 6–24 months of age who were not toilet trained. Urine was collected by applying pediatric urine bags to the children during office or home visits (Eskenazi et al. 2003). When children were not able to produce a void during scheduled contacts, study staff trained parents to apply the urine bag at home and to then place the urine in a clean cup provided to them. The parent was instructed to call the field office as soon as the void was produced, and study staff then retrieved the sample.

Cotton inserts have also been used to recover urine from diapers (Hu et al. 2000). However, the most promising development for sampling infants and toddlers who are not yet toilet trained appears to be extracting the metabolites from the diaper gel matrix, although this method still needs to be evaluated for multiple groups of pesticides (Hu et al. 2004).

### Preschool children’s exposure.

Urine samples have been collected in nearly all studies of pesticide exposure among preschool children. Urine samples have been analyzed for common metabolites, such as the dialkylphosphate (DAP) compounds or for compound-specific metabolites [e.g., 3,5,6-trichloro-2-pyridinol (TCPy) for chlorpyrifos]. Major exposure assessment issues of concern are duration of collection (spot samples vs. 24-hr samples) and frequency of sampling.

Collection of single urine voids, often referred to as spot urine samples, has been selected as a primary sampling strategy for several practical reasons. The burden it places on study participants is relatively low, and sample processing and analysis are manageable and affordable. However, several studies have now determined that pesticide metabolite concentrations in children’s spot urine samples can exhibit high intraindividual (within-child) variability (Adgate et al. 2001; Koch et al. 2002). In studies in which it is possible to collect only a single urine sample per day, the first morning void is preferred, because the urine is more concentrated, the collection period is longer (usually > 8 hr), and it appears this sample is most representative of the daily average (Kissel et al. 2005). Collection of repeated spot urine samples during a single day or over several days is one means of addressing the issue of intraindividual variability. These repeated measures can be averaged to produce a more stable estimate of exposure and would allow evaluation of exposures during specific windows of vulnerability.

Collection of complete 24-hr urine samples has become a standard part of many occupational exposure studies but has generally been viewed as impractical for small children. Several studies reviewed here have attempted to collect 24-hr samples but have been only partially successful. A recent study (Kissel et al. 2005) of 25 children in a low-income, low-literacy population by the Berkeley center provided intensive training of participants, detailed record keeping by participants, use of small refrigerators, and daily contact by research staff to improve compliance; it was estimated that 28% of participants provided complete samples, an additional 12% were likely complete, 52% missed one or two voids, and 8% likely missed more than two voids.

Several of the centers have also collected blood samples from children postnatally. The Columbia center has employed a pediatric phlebotomist to draw blood when children came to the center for the developmental assessment. Samples were collected from 98% of the children that were seen. However, volumes were generally low (an average of 6.8 mL collected at 24 months and 6.2 at 36 months). The Berkeley center hired a pediatric phlebotomist to collect blood for both state-required lead screening and the CHAMACOS (Center for Health Analysis of Mothers and Children of Salinas) study, increasing the rate of blood collection. Repeat blood samples can be collected from young children but are more difficult to obtain than are urine samples.

Children’s activities are an important variable in assessing pesticide exposure. The Berkeley center has used a visually based, low-literacy child activity time line for parents to record child activity and location. The University of Washington center and the PNASH center have employed miniaturized global positioning system (GPS) units to produce detailed documentation of children’s time–location patterns (Elgethun et al. 2003). Recent studies have found that time–location diaries kept by parents produce relatively poor agreement with the GPS measurements, suggesting that such diary data would result in substantial misclassification. The GPS analysis has also shown that transient peak exposures can occur both temporally and spatially and that such exposures are not adequately captured within the resolution of most microenvironmental analysis studies.

### School-age children exposure.

Sampling procedures for school-age children are similar to those described above for preschool children. However, as children reach school age, they are more likely to be able to participate more actively in studies. They may be able to assent to study procedures, wear personal sampling devices, collect more complete urine samples, and provide helpful information regarding pesticide sources and their own activities. Here we would stress greater emphasis on personal sampling devices to improve the quality of exposure data for this age group.

### Saliva monitoring.

The PNASH center has explored the feasibility of saliva sampling for pesticides in both workers and children (Denovan et al. 2000; Lu et al. 2003). Current saliva sample collection methods require that children chew on a cotton or synthetic plug for approximately 2 min. The plug, containing up to 2 mL of saliva, is then placed in a vial for storage. The plug is similar in size to a dental sponge and could pose a choking hazard to children < 3 years of age. The Berkeley center has experimented with pipettes to directly transfer saliva from a child’s mouth into a collection container. Sample volumes, however, have been < 1 mL. In rare cases, children have spit directly into a beaker. It is not clear that these techniques provide an adequate or appropriate saliva sample for pesticide analysis.

### Participation of cohort members in environmental and biologic sampling.

Collection of an array of biologic and environmental samples from women during pregnancy and soon after birth places a burden on study participants and may lead to attrition regarding participation in the exposure assessment component of these studies. Tables 3 and 4 provide data from the birth cohort studies reviewed here to indicate what might be anticipated in the National Children’s Study. Sample sizes are presented for each study and for each relevant time category; the percentage of enrolled study members is then provided for each of the biologic or environmental samples. It is important to recognize that not all of the rates in Tables 3 and 4 are directly comparable. For example, the Berkeley study accepted all eligible enrollees with no condition that they participate in every exposure assessment event; in contrast, enrollment criteria for the Columbia study included collection of a cord blood sample from each participant at delivery. Participation in environmental and biologic sampling tends to drop over time and can be relatively low for certain types of samples. Factors contributing to low participation include reliance on delivery staff, emergency deliveries, inability to schedule appointments that include both parents, mobile populations that are hard to track, and the absence of children from the home at the time of visits by study staff. Participation can also be enhanced; for example, the Berkeley center saw an increase from 64 to 81% between 12 and 24 months for child blood samples because of the hiring of a child phlebotomist who went to each home.

## Challenges in the Analysis of Pesticide Exposure Samples

Increased interest in children’s exposure to pesticides has resulted in the generation of large numbers of samples for analysis. In this section we discuss several key issues and lessons learned regarding analysis.

### Laboratory capacity.

As studies of the type described here grow larger and a series of longitudinal samples are collected from each participant, the sample size may become too large for the capacity of one or two laboratories. Multiple laboratories should be enlisted for large studies to avoid sample backlogs. As laboratory capacity is improved, it is imperative to produce comparable data across studies, as the U.S. EPA did in its interlaboratory comparison study among the North American laboratories performing DAP analyses (James et al. 2003).

### Intra- and interpersonal variability in urine samples.

Several methods have been evaluated to “correct” for the variability in urine dilution across spot samples, the most popular being creatinine (Boeniger et al. 1993). Creatinine excretion varies because of many factors, including the size of the participant, so interindividual variation, especially among diverse populations, is large. Thus, creatinine-adjusted pesticide concentrations should never be compared among individuals of vastly different age groups (i.e., children vs. adults). Changes in creatinine excretion during pregnancy should be thoroughly evaluated before comparing with other women in similar age groups. The validity of creatinine adjustment may also be analyte dependent. Further studies to assess the variability of commonly measured analytes in urine should be conducted to identify the most effective sampling strategies for cohort studies. In all likelihood, sampling for nonpersistent chemicals will require multiple samples taken over the course of the study at regular intervals (e.g., weekly, monthly, semiannually).

### Selectivity of analysis.

Selectivity can refer to either the ability of a measurement technique to differentiate a single analyte that is measured from other components of the matrix (i.e., reducing false positives) or the ability of the analyte measured to accurately, and unequivocally, identify exposure to the target chemical of interest. However, high selectivity techniques are costly and require specialized training for operation (Barr et al. 1999). Methods such as immunoassays and less specialized technologies may be employed, but harmonization should be performed to ensure that data generated using different methods are comparable.

The selectivity of the analyte measured to accurately reflect the exposure of interest may depend on the biomarker being measured rather than the measurement technique. Many OP pesticides, for example, can be metabolized to common DAP compounds, so it is not possible to derive chemical-specific exposure estimates from such data. Further complicating the issue, the DAPs, as well as compound-specific metabolites, may be present in environmental media as the environmental degradates of the pesticides (Curl et al. 2003b; Wilson et al. 2004). No studies to date have shown that these environmental degradates can be absorbed and excreted unchanged; but if this does occur, then DAPs and other pesticide metabolites detected in urine would represent exposure to both the pesticide and its degradate. Some metabolites are very selective for the chemical measured. For example, 2-isopropoxy-4-methyl-6-hydroxypyrimidine, a metabolite of diazinon, is selective for diazinon exposure, although potentially the environmental degradates could contribute to the urinary levels as well. In some cases, the parent pesticide can be excreted in urine, such as for the herbicide 2,4-D (2,4-dichlorophenoxy-acetic acid).

One way to unequivocally identify exposure to a particular pesticide is by measuring the intact pesticide, presumably in blood or similar samples, because the intact pesticide is not appreciable in urine. However, blood measurement levels are typically about 1,000 times lower than urinary metabolite measurements; this requires highly sensitive analytical techniques, driving up the cost of analysis. In addition, target chemicals in blood may exhibit some degree of instability. Finally, there are no laboratory methods available for many common use agricultural or home pesticides in blood. Saliva sampling is an attractive alternative to blood sampling, as discussed above.

### Sensitivity of analysis.

The sensitivity of an analytical method—the ability of the method to measure the chemical at the desired level—should be considered before a study begins (Barr et al. 1999). The biologic half-lives of nonpersistent chemicals are relatively short, usually on the order of hours or days (Needham and Sexton 2000). Samples collected several days after an exposure event may require ultra-sensitive methods for analyte detection. These measurements must provide adequate sensitivity to allow detection of the chemicals of interest in a sufficient proportion of the population to provide a realistic representation of the populations’ exposure. The current method for analysis of OP pesticide metabolites developed by the Centers for Disease Control and Prevention was used for many but not all of the studies described in this article and has proven to be quite sensitive (Bravo et al. 2002).

### Alternative matrices and/or biomarkers.

Pesticides have been measured successfully in saliva (Lu et al. 2003), meconium (Whyatt and Barr 2001), and amniotic fluid (Bradman et al. 2003). Matrices such as meconium may provide longer term dosimeters for exposure to nonpersistent chemicals; saliva may provide a measure of internal dose without the invasiveness of blood sampling. Preliminary studies evaluating the partitioning of chemicals in the various matrices should be conducted that will allow for comparison of data among matrices and validate the usefulness of alternative matrices for biologic monitoring. An alternative matrix that may prove useful is the gel matrix in disposable diapers. Extraction techniques for solid materials may prove practical for the gel matrix and might improve sample collection procedures for infants and children who are not toilet trained.

### Quality assurance and control.

A vital component of all biomonitoring methodology is a sound quality assurance/quality control (QA/QC) program. QA/QC procedures supporting these studies have included proficiency testing, repeat measurements of known biologic materials, and round-robin studies to confirm reproducible measurement values among laboratories, as well as field spikes and field blanks to confirm sample integrity.

### Sample storage issues.

The time frame for sample testing and long-term storage becomes an issue for large studies. The long-term stability of analytes has been demonstrated for some matrices but not for others, for example, blood. One final logistical complexity is physical freezer space for storage, and the substantial cost of maintaining that storage. Archiving samples in the smallest containers possible would enhance the ability to keep the samples long term under proper storage conditions.

## Conclusions

Epidemiologic investigations have often relied on questionnaire information for exposure classification, but this approach alone is unlikely to capture the complexity of children’s pesticide exposure. In contrast to the Agricultural Health Study, for example, which draws on the records of pesticide applicators and has derived a complex exposure algorithm from 40 years of occupational exposure studies (Dosemeci et al. 2002), the everyday use of pesticides in homes, schools, and other child environments is not easily codified, and dietary pesticide exposures can only be inferred from questionnaire data. It seems, therefore, that some level of environmental and/or biologic monitoring will be required for all study participants. The type of sampling needed will depend primarily on the purpose of the study, be it exposure characterization, long-term health outcomes, or short-term toxic response in children. Lessons learned regarding pesticide exposure can be summarized as follows:

- Biologic monitoring appears to be the best available method for assessment of children’s exposure to pesticides. However, all pesticide biomarkers have limitations. It is likely that a combination of biomarkers, environmental measurements, and questionnaires will be needed after careful consideration of the specific hypotheses posed by investigators and the limitations of each exposure metric.
- Environmental measurements, such as surface wipes and indoor air or house dust samples, can characterize residential pesticide contamination, but their validity for exposure classification has not been established. Their value in epidemiologic studies deserves further investigation.
- Emphasis on personal rather than environmental sampling in conjunction with urine or blood sampling is likely to be most effective at classifying exposure.
- A focus on maternal exposures during pregnancy is particularly important for making associations with infant health, given the critical developmental stages during this period.
- Questionnaires will need to be validated with carefully designed studies that involve personal sampling or biologic monitoring.
- Interpretation of urinary metabolites is not straightforward, but because of ease of collection, these samples may provide the best available approach to capturing exposure variability of nonpersistent pesticides in young children; additional validation studies are needed.
- Repeated exposure measures will be needed to overcome high intraindividual variability of biologic samples for most pesticides in use today.
- Postnatal exposure can also contribute to health effects in early childhood. For infants and young children, it appears possible to collect urine samples for extended periods of time.
- Expansion of laboratory capacity will require careful attention to QA/QC and will need to include formal procedures for ensuring inter-laboratory comparability in sample analysis.
- Saliva measurements of pesticides, if feasible, would overcome the limitations of urinary metabolite-based exposure analysis.
- GPS technology appears promising in the delineation of children’s time–location patterns.

It is clear from this review that the critical tools needed for accurate characterization of children’s pesticide exposure are not yet in place. Most of the work discussed here has been conducted in the past 6–8 years, and many of the exposure methods have been exploratory in nature. Substantial resources will be needed for validation of existing methods, support of novel methods, and enhancement of analytical capabilities. It may be possible to initiate epidemiologic investigations and validation studies simultaneously, if biomarker samples can be properly archived. Whatever sampling strategies are employed for epidemiologic investigations, they will need to be selected to support specific hypotheses and focus on specific pesticides. Studies with substantial exposure assessment activities will be costly but should ultimately pay benefits in terms of the quality of scientific information produced.

## Figures and Tables

**Table 1:** Exposure data collected in reviewed studies of children’s exposure to pesticides through 2003.

Study^a^	Pesticide source information	Environmental monitoring	Maternal exposure	Perinatal/infant exposure	Preschool children	School-age children
Columbia University Birth cohort study	Residential pesticide use	Indoor air	Blood, urine, personal air	Cord blood, urine, meconium	Blood, urine	Blood, urine
Columbia University Prenatal intervention study	Residential pesticide use	Indoor air	Blood, urine	Cord blood		
University of California at Berkeley Birth cohort study	California Pesticide Use reports, home inventory,^b^ proximity to agricultural spray,^c^ parental work^d^	House dust, vehicle dust	Blood, urine, breast milk	Cord blood, blood, urine	Blood, urine, saliva	Blood, urine, saliva
University of California at Berkeley Specialized studies	California Pesticide use reports, home inventory, proximity of agricultural spray, parental work	Indoor/outdoor air, house dust, surface wipe^f^	—	Diaper and spot urine, amniotic fluid, surface wipe^f^ duplicate diet	1st morning void, 24 hr urine, saliva,^e^ CAT^g^	—
Mount Sinai Medical Center Birth cohort study	Residential pesticide use	—	Blood, urine	Cord blood, urine	Urine	—
Mount Sinai Medical Center Community cohort	Cockroach enumeration^h^	Indoor air, house dust, surface wipes	Urine	Urine, hand wipes^i^	Urine, hand wipes	—
University of Washington Community intervention	Residential pesticide use, parental work	House dust	—	—	Urine	—
University of Washington Community intervention	Residential pesticide use, parental work, proximity to agricultural spray	House dust, vehicle dust	—	—	Urine	—
University of Washington Spray drift exposure	Residential pesticide use, aerial application^j^	Indoor/outdoor air, residential surfaces, outdoor deposition	—	—	Hand wipes, personal GPS^k^	Hand wipes, personal GPS
PNASH center Agricultural families	Residential pesticide use, parental work, proximity to spray	—	—	—	Urine	—
PNASH center Aggregate exposure	Residential pesticide use, home inventory, diet diaries^l^	Indoor air, house dust, surface wipes, duplicate dietm	—	—	Urine, hand wipes	—
PNASH center Longitudinal exposure	Residential pesticide use, parental work, proximity to agricultural spray	—	—	—	Urine	—

— , no data.

a References for studies: Columbia University (Carlton et al. 2004; Perera et al. 2003; Whyatt and Barr 2001; Whyatt et al. 2002, 2003, 2004; Berkeley center (Bradman et al. 2003; Castorina et al. 2003; Eskenazi et al. 2003, 2004; Goldman et al. 2004); Mount Sinai center (Berkowitz et al. 2003, 2004; Brenner et al. 2003); University of Washington center (Curl et al. 2002; Elgethun et al. 2003); PNASH center (Fenske et al. 2002a, 2002b; Kissel et al. 2005; Koch et al. 2002; Lu et al. 2000, 2001, 2004; Simcox et al. 1995).

b Home inventory: visual inspection of pesticide products currently in the residence, along with detailed history of pesticide use.

c Proximity to agricultural spray: normally defined as distance between residence and nearest pesticide-treated farmland; more refined analyses include meteorologic data and pesticide application history; determined by GPS technology.

d Parental work: parent or other household member works in agriculture in a job with potential pesticide exposure.

e See Denovan et al. (2000).

f Surface wipe samples in this study included press samples using the modified Edwards-Lioy sampler.

g CAT: child activity time line, developed as a visual, low-literacy diary for child location and activity.

h Cockroach enumeration: conducted before and after integrated pest management (IPM) activities to determine effectiveness of intervention.

i Hand wipes: children’s hands wiped or rinsed with isopropanol solution; requires skin removal efficiency information for interpretation.

j Aerial application: data on application rates, frequency, and duration of commercial pesticide applications near study community.

k Personal GPS: portable GPS units with data-logging capability suitable for studies of small children (Elgethun et al. 2003).

l Diet diaries: 3-day parental diary of all fresh produce (fruits and vegetables) and juices consumed by child, classified as either organic or conventional foods.

n Duplicate diet: representative portions of all foods consumed by child in a 24-hr period.

**Table 2:** Measured analytes in five biologic sample matrices.^a^

Study	Maternal blood	Cord blood	Child blood	Maternal urine	Child Urine
Columbia University center	OP insecticides, carbamate insecticides, pyrethroid insecticides, herbicides, fungicides, diethyltoluamide, organochlorine insecticides, PCBs, PAH-DNA, antioxidants, cotinine	OP insecticides, carbamate insecticides, pyrethroid insecticides, herbicides, fungicides, diethyltoluamide, organochlorine insecticides, PCBs, PAH-DNA, antioxidants, cotinine, lead, mercury	OP insecticides, carbamate insecticides, pyrethroid insecticides, herbicides, fungicides, diethyltoluamide, organochlorine insecticides, PCBs, PAH-DNA, antioxidants, cotinine	OP DAP metabolites, specific OP metabolites, carbamate metabolites, pyrethroid metabolites, herbicides, other	Collected at 36 and 60 months; stored for future analysis
University of California at Berkeley center	Organochlorine insecticides, cholinesterase, PCBs, PON1 status, PBDEs (subset)	Organochlorine insecticides, lead, cholinesterase, PCBs, PON1 status	Lead	OP DAP metabolites, OP-specific metabolites, carbamate metabolites, pyrethroid metabolites, herbicides, other	OP DA metabolites, OP-specific metabolites
Mount Sinai Medical Center	Organochlorine insecticides, cholinesterase, paraoxonase, PCBs, lead	Cholinesterase, lead, paraoxonase	Not collected	OP DAP metabolites, OP-specific metabolites, pentachlorophenol, pyrethroid metabolites	Collected; not yet analyzed
University of Washington center	Not collected	Not collected	Not collected	OP DAP metabolites	OP DAP metabolites, OP-specific metabolites
PNASH center (University of Washington)	Not collected	Not collected	Not collected	Not collected	OP DAP metabolites, OP-specific metabolites

Abbreviations: DAP, dialkylphosphate; PAH, polyaromatic hydrocarbon; PBDE, polybrominated diphenyl ether; PCB, polychlorinated biphenyl; PON1, paraoxonase .

a Specific analytes for chemical classes are as follows: OP insecticides (blood): chlorpyrifos, diazinon, dichlorvos, fonophos, malathion, methyl parathion, parathion, phorate, terbufos; carbamate insecticides and metabolites (blood): bendiocarb, carbofuran, propoxur, 2-isopropoxyphenol (propoxur metabolite), carbofuranphenol (carbofuran metabolite), 1-naphthol (naphthalene and carbaryl metabolite); pyrethroid insecticides (blood): trans-permethrin, cis-permethrin; herbicides (blood): acetochlor, alachlor, atrazine, chlorthal-dimethyl, metolachlor, trifluralin; fungicides (blood): chlorthalonil, dicloran, metalaxyl, captan metabolite, folpet metabolite; organochlorine insecticides (blood): p,p-dichlorodiphenyldichloroethylene, p,p-dichlorodiphenyltrichloroethane, o,p-dichlorodiphenyltrichloroethane, dieldrin, heptachlor epoxide, hexachlorobenzene, β/γ-hexachlorobenzene, mirex, oxychlordane, trans-nonachlor; OP DAP metabolites (urine): dimethylphosphate, dimethylthiophosphate, dimethyldithiophosphate, diethylphosphate, diethylthiophosphate, diethyldithiophosphate; OP-specific metabolites (urine): 3,5,6-trichloropyridinol (methyl/ethyl chlorpyrifos), 4-nitrophenol (methyl/ethyl parathion, ethyl p-nitrophenylbenzenethiophosphonate), malathion dicarboxylic acid, acephate, methamidaphos (acephate, methamidaphos), 2-isopropyl-4-methyl-6-hydroxypyrimidine (diazinon), hydroxycoumarin (coumaphos), pirimiphos methyl metabolite, isazaphos methyl metabolite, o-methoate, dimethoate; carbamate metabolites (urine): 2-isopropoxyphenol (propoxur metabolite), carbofuranphenol (carbofuran metabolite), 1-naphthol (naphthalene and carbaryl metabolite); pyrethroid metabolites (urine): 3-phenoxybenzoic acid, cis/trans-dichlorodimethylvinyl cyclopropane carboxylic acid, cis-dibromodimethylvinyl cyclopropane carboxylic acid, 4-phenoxybenzoic acid; herbicides or metabolites (urine): alachlor mercapturate, atrazine mercapturate, acetochlor mercapturate, 2,4-D, metolachlor mercapturate; others: o-phenylphenol, pentachlorophenol, 2,4-dichlorophenol, 2,5-dichlorophenol (paradichlorobenzene metabolite), 2,4,5-trichlorophenol, 2,4,6-trichlorophenol, ethylene thiourea, propylene thiourea.

**Table 3:** Percent participation of cohort members in biologic sampling procedures in four birth cohort studies.^a^

Study	Baseline	26 weeks gestation	Delivery	6 months	12 months	24 months
University of California at Berkeley birth cohort^b^	n = 528	n = 528	n = 528	n = 477^c^	n = 445^c^	n = 425^c^
Maternal urine	99	94	94	93^d^	—	—
Paternal urine	—	—	51	—	—	—
Child urine	—	—	—	88	91	90
Maternal blood	—	81	—	—	—	—
Cord blood	—	—	74	—	—	—
Child blood	—	—	—	—	64	81
Breast milk	—	—	63	93^d^	—	—
Mount Sinai Medical Center birth cohort^b^	n = 479	—	n = 404^e^	—	n = 215	n = 305
Maternal urine	91	—	—	—	—	—
Child urine	—	—	—	—	100	94
Maternal blood	90	—	—	—	—	—
Cord blood	—	—	59	—	—	—
Mount Sinai Medical Center IPM cohort^b^	n = 184	—	—	—	n = 112	n = 56^f^
Maternal urine	98	—	—	—	96	100
Child urine	—	—	—	—	84	82
Columbia University birth cohort^b^	n = 588^g^		n = 588^g^			n = 449^h^
Maternal urine	82^i^	—	—	—	—	—
Maternal blood	—	—	99	—	—	—
Cord blood	—	—	81	—	—	—
Meconium	—	—	51^j^	—	—	—
Child blood	—	—	—	—	—	71

— , no samples collected at those time periods.

a Percentages are calculated based on sample size provided for each study and time category; percent participation values are for participation in the biologic sampling procedures only and do not reflect retention rates for the cohorts.

b Berkeley cohort (CHAMACOS) recruited in Salinas Valley, California: n = 528 based on live births; total enrolled = 601 (Eskenazi et al. 2003, 2004). Mount Sinai birth cohort of primiparous pregnant women enrolled 1998–2003 (Berkowitz et al. 2003); Mount Sinai IPM cohort (Growing Up Healthy Integrated Pest Management Cohort; Brenner et al. 2003). Columbia birth cohort (Whyatt et al. 2003).

c Based on number of mothers participating rather than children due to several cases of twins.

d Based on number of women breast-feeding 6 months postpartum.

e Severnty-five women were excluded from follow-up for medical complications, very premature births (< 32 weeks gestation or < 1,500 g), delivery of an infant with birth defects, inability to obtain biologic specimens before delivery, change of residence, or refusal to continue to participate.

f Number of participants reached through the end of October 2003.

g Fully enrolled; subjects are considered fully enrolled once the prenatal monitorings and questionnaires had been completed and blood samples (from the mother and/or newborn) had been collected at delivery.

h Number of subjects currently enrolled at the time of the scheduled assessment whether or not the assessment was completed; subjects are dropped from the cohort if no contact is made for 1 year from the last scheduled assessment.

i A single urine sample is being collected from the mothers during pregnancy and is being stored for future analyses. Biweekly urine samples are being collected on a subset of 100 women beginning during the 32nd week of pregnancy through delivery and are being analyzed as indicated in Table 4.

j Collected from a subset of newborns under supplemental funding from the U.S. EPA STAR grant program.

**Table 4:** Percent participation of cohort members in biologic sampling procedures in three birth cohort studies.^a^

Study	Prenatal	6 months	12 months	24 months
University of California at Berkeley birth cohort^c^	n = 528	n = 473^b^	n = 442^b^	n = 422^b^
Home inspection/house dust	91	81	86	88^d^
Mount Sinai Medical Center IPM cohort^c^	n = 184	—	n = 112	n = 56
Air sample	100	—	100	100
Hand wipe	50	—	92	100
Toy wipe	75	—	96	100
Dust	96	—	100	100
Columbia University birth cohort^c^	n = 588			
48-hr personal air	100	—	—	—
2-week integrated indoor air	17^e^	—	—	—
Kitchen dust samples	17^e^	—	—	—

— , no samples collected at those time periods.

a Percentages are calculated based on sample size provided for each study and time category; percent participation values are for participation in the environmental sampling procedures only and do not reflect retention rates for the cohorts.

b Percent participation at 6, 12, and 24 months based on number of mothers participating rather than children due to several cases of twins.

c Berkeley cohort (CHAMACOS) recruited in Salinas Valley, California (Eskenazi et al. 2003, 2004); Mount Sinai IPM cohort (Growing Up Health Integrated Pest Management Cohort, 1999–2002; Brenner et al. 2003); Columbia birth cohort (Whyatt et al. 2003).

d Percentage permitting home visits at 24 months; no house dust collected.

e Collected from a subset of 100 homes beginning during the 32nd week of pregnancy and continuing through delivery; kitchen dust samples are also collected from a subset of homes.
